# Reactive oxygen species (ROS) in cancer: from redox signaling and metabolic plasticity to therapeutic vulnerabilities

**DOI:** 10.37349/etat.2026.1002383

**Published:** 2026-07-23

**Authors:** Taslim Uddin, Tajmin Khanam, Afia Asma, Mayesha Tasnim, Tanbin Sultana, Nafisa Parvez Aruba, Mohammad Hasan, Anika Zaheen, Anika Tabassum Aziz, Abhishek Karmaker Joy, Nazifa Anjum, Nusrat Jahan, Rhea Sarkar Nipun, Sushmita Sharma, Maisha Maliha Misha, Most. Tamanna Haque, Shubhro Saha

**Affiliations:** Fondazione Policlinico Universitario Agostino Gemelli IRCCS, Italy; ^1^Department of Biotechnology and Genetic Engineering, Jahangirnagar University, Savar, Dhaka 1342, Bangladesh; ^2^National Institute of Textile Engineering and Research (NITER), University of Dhaka, Dhaka 1000, Bangladesh; ^3^Department of Neurology, Chittagong Medical College, Chattogram 4203, Bangladesh; ^4^Master in Public Health, National Institute of Preventive and Social Medicine (NIPSOM), Dhaka 1212, Bangladesh; ^5^Department of Biochemistry and Biotechnology, University of Science & Technology Chittagong, Chattogram 4202, Bangladesh; ^6^Department of Biochemistry and Biotechnology, North South University, Dhaka 1229, Bangladesh; ^7^Department of Biomedical Engineering, Bangladesh University of Engineering and Technology, Dhaka 1205, Bangladesh; ^8^Department of Community Medicine, Dhaka Medical College, Dhaka 1000, Bangladesh; ^9^Sir Salimullah Medical College, Dhaka 1100, Bangladesh; ^10^Department of Medicine, Sir Salimullah Medical College & Mitford Hospital, Dhaka 1100, Bangladesh; ^11^Department of Food & Nutrition, GCAHS, University of Dhaka, Dhaka 1205, Bangladesh; ^12^Rajshahi Medical College, Rajshahi 6100, Bangladesh; ^13^MRes Public Health, University of Wolverhampton, Wolverhampton WV1 1LY, UK

**Keywords:** ROS, oxidative stress, redox signaling, cancer metabolism, tumor microenvironment, targeted therapy

## Abstract

Reactive oxygen species (ROS) are important regulators of cancer biology, acting as tumor-promoting signaling mediators and inducers of oxidative cell death. Oncogenic signaling, mitochondrial dysfunction, metabolic rewiring, and microenvironmental stress lead to increased basal ROS levels in cancer cells, resulting in a state of chronic oxidative pressure. Tumors develop adaptive antioxidant programs such as glutathione and thioredoxin, NADPH regeneration pathways, and sustained activation of the Nrf2–Keap1 axis to adapt to these conditions, leading to redox plasticity and “Nrf2 addiction” in some cancers. This adaptive rewiring allows malignant cells to sustain proliferative signaling while evading ROS-induced cytotoxicity and contributes substantially to therapeutic resistance. Despite the great promise of ROS-targeted therapies in preclinical studies, their translation into the clinic has been challenging for decades. Large antioxidant trials failed or even increased cancer risk. Many pro-oxidant therapies have limited efficacy due to a narrow therapeutic window, systemic toxicity, poor tumor selectivity, and a dynamic ability of tumors to reprogram antioxidant defenses. The significant intra-tumoral and spatial heterogeneity of redox status further complicates these constraints, where different tumor regions and cellular subpopulations exhibit different metabolic states, ROS thresholds, and sensitivities to ferroptosis. Emerging evidence indicates that ferroptosis, an iron-dependent cell death triggered by lipid peroxidation, is a significant therapeutic liability of redox-adapted tumors, particularly when antioxidant buffering systems like GPX4, system Xc^–^, FSP1, or DHODH are impaired. This review discusses the molecular functions of ROS in tumor initiation, progression, immune regulation, metabolic adaptation, and therapeutic resistance and critically analyzes the reasons for clinical challenges in redox-targeted interventions despite extensive research. The review highlights the importance of adaptive antioxidant rewiring, redox-dependent metabolic flexibility, and the complexity of the tumor microenvironment in determining the therapeutic outcome. Finally, novel strategies in precision redox oncology are discussed, including biomarker-driven patient stratification, real-time redox profiling, ferroptosis-targeted therapies, and rational combination approaches with the aim to exploit tumor-specific redox vulnerabilities while minimizing toxicity to healthy tissues.

## Introduction

Reactive oxygen species (ROS) refer to a collection of highly reactive substances that incorporate oxygen, including free radicals such as superoxide (O_2_•^–^) and the hydroxyl radical (•OH), as well as nonradicals such as hydrogen peroxide (H_2_O_2_). Although ROS are usually discussed in the context of oxidative stress and cell damage, they can also serve as important signaling molecules that control a range of cellular activities, such as cell proliferation, differentiation, immune response, and apoptosis [1]. ROS are mainly generated as byproducts of mitochondrial respiration that occurs within the inner mitochondrial membrane during aerobic metabolisms. ROS are important for cellular redox homeostasis and act as secondary messengers in various signaling pathways at low to moderate levels [2, 3]. ROS in cancer should not be viewed simplistically as merely beneficial or harmful, but rather as highly compartmentalized, threshold-dependent, and context-specific signaling mediators whose biological effects depend on concentration, subcellular localization, and redox buffering capacity [4, 5]. Recent advances in redox biology have transformed ROS from generic harmful byproducts to highly segregated and finely regulated signaling mediators. Due to source, diffusion capacity, and local antioxidant buffering systems, mitochondrial ROS (mtROS) and cytosolic ROS have different physiologic impacts on ROS signaling. ROS can specifically regulate signaling pathways without causing broad oxidative damage due to redox compartmentalization. ROS signaling selectivity is achieved by reversible oxidation of cysteine residues in redox-sensitive proteins, which modulates phosphatases, kinases, transcription factors, and metabolic enzymes geographically and temporally [6–8]. At low to moderate levels, ROS can activate various oncogenic pathways, such as PI3K/AKT, MAPK, and NF-kB, which control key processes, such as proliferation, invasion, and immune evasion [9]. On the contrary, excessive accumulation of ROS surpasses the antioxidant buffering capacity of cells leading to oxidative damage, genomic instability and activation of regulated cell death pathways including apoptosis, ferroptosis and so on. Importantly, the functional consequence of ROS is not solely dependent on their concentration, but also on their subcellular localization and cellular redox buffering capacity, maintained by systems such as glutathione (GSH), thioredoxin (Trx) and NADPH [10–12]. ROS modulates p53 activity in a context-dependent manner. Under moderate oxidative stress, ROS-induced DNA damage activates ATM/ATR signaling, leading to p53 stabilization and the induction of cell cycle arrest, DNA repair, or apoptosis. In contrast, sustained or excessive ROS can impair p53 function through oxidative modification of its DNA-binding domain or by promoting mutagenesis, thereby contributing to tumor progression [13, 14].

In addition to their direct effects on tumor cell survival and genomic instability, ROS are important regulators of immune-cell function within the tumor microenvironment (TME). Accumulating evidence suggests that oxidative stress regulates antitumor immunity in a context-dependent manner by regulating dendritic cell maturation, macrophage polarization, T-cell exhaustion, immune checkpoint signaling, and cytokine production. ROS are not only damaging metabolic by-products but also serve as dynamic immunoregulatory mediators that shape tumor progression, immune evasion, and therapeutic responsiveness [15–17]. The high levels of ROS that are associated with cancer progression are also what expose the cancer cells to other modes of therapy that cause an increase in the levels of oxidative stress. Targeted ROS-based cancer therapies are expected to exploit this weakness by enhancing ROS production in cancer cells, thereby causing cell death through oxidative injury to DNA, lipids, and proteins [18, 19]. ROS-targeted therapies offer medical potential, but several issues make them difficult to employ. For example, selectivity is a major issue. Due to chronic oxidative stress, cancer cells may activate adaptive responses that render them resistant to increased ROS production. For example, cancer cells can stimulate ROS-sensitive signaling pathways, including NF-kB and PI3K/AKT, which promote cell survival, proliferation, and apoptotic resistance [20]. ROS-based cancer therapies may also be resisted by cancer cells through increased expression of antioxidant defense mechanisms, including Nrf2 and GSH, which counteract excess ROS and prevent oxidative cell damage [21, 22]. Besides, another problem is the heterogeneity of tumors in that various types of cancer cells have different amounts of ROS generation and antioxidants which poses a challenge for developing a universal ROS-targeted treatment [23, 24]. Thus, it is necessary to develop individualized ROS-based treatments, in which tumor vulnerability to ROS can be measured and addressed accordingly [25, 26].

Although several investigations and studies have been done on the use of ROS in cancer treatment, several research gaps remain to fully exploit ROS in treating cancer cells. Among the significant gaps, the knowledge of the exact molecular pathway by which ROS are involved in the TME and immune modulation is lacking [27, 28]. The ROS signaling pathways interact with other intracellular networks, such as DNA repair, cell survival, metastasis, and inflammation. Greater insight into how ROS regulates these pathways will aid in identifying specific therapeutic targets [29, 30]. To address the obstacles associated with ROS-targeted therapies, several mitigation measures have been proposed. The first method is the combination of ROS-inducing agents with the conventional therapies, including chemotherapy, radiation, and immunotherapy [19, 31]. The approach exploits the synergistic effects of ROS-induced cytotoxicity, increases the effectiveness of traditional interventions, and circumvents the effects of resistance strategies [32, 33]. For example, radiation therapy produces ROS that can be combined with ROS-modulating agents to enhance the therapeutic effect of radiation in cancer cells. It is possible to design nanoparticles and liposomal formulations that selectively target cancer cells with prooxidant drugs or ROS modulators, thereby reducing the toxicity of normal tissues and enhancing therapeutic effect, indicating another potential way [18, 34]. In addition, biomarker-directed strategies that can identify tumors highly susceptible to ROS can be used to select the right patients and enhance cancer-specific therapies [19, 25].

This review aims to provide a comprehensive overview of the dual role of ROS in cancer, highlighting their involvement in tumor progression, metabolic reprogramming, and therapy resistance. It further explores emerging redox-targeted therapeutic strategies and emphasizes the need for precision modulation of ROS to exploit cancer-specific vulnerabilities for improved clinical outcomes.

## ROS in cancer: their origins and regulatory mechanisms

### Generation of ROS in mitochondria

ROS mostly come from the electron transport chain (ETC) in the mitochondria. There is a lot of superoxide anion made by the ETC. Five protein groups, called complexes I through V, make up the ETC, and they work together to make ATP [35, 36]. During oxidative phosphorylation, superoxide is made when electrons leave complexes I and III. mtROS are primarily generated at complexes I and III of the ETC [37]. Their contributions fluctuate based on factors such as metabolic state, substrate availability, oxygen tension, mitochondrial membrane potential, and the direction of electron flux. Complex I serves as a significant source of superoxide production, particularly under conditions that promote reverse electron transport (RET), such as succinate accumulation and elevated membrane potential or depolarization. Conversely, complex III can also contribute notably to ROS generation in specific physiological and pathological contexts, primarily due to electron leakage. Consequently, the formation of mtROS cannot be solely linked to a particular respiratory complex; rather, it is a dynamic process influenced by various conditions [38–40]. When an electron from NADH is transferred to flavin mononucleotide (FMN) and subsequently overflows to O_2_, or when reduced flavin mononucleotide (FMNH_2_) contributes an electron to O_2_, the hydrophilic region of complex I may produce superoxide anion. Complex III (ubiquinol-cytochrome c oxidoreductase) receives reducing equivalents generated by complexes I and II and employs the Q-cycle mechanism for their degradation [41]. Genetic and acquired abnormalities in the respiratory chain of the mitochondria may cause complex III to overproduce ROS. Therefore, it is yet unknown how complex III might contribute to the generation of gross mtROS in steady-state physiological settings [42].

Mitochondria also generate spatially restricted ROS nanodomains that allow local redox signaling, which can selectively modify nearby proteins without causing excessive oxidative stress. This spatial specificity is tightly associated with mitochondrial dynamics, including a constant cycle of fission and fusion, which regulates organelle morphology, bioenergetic efficiency and ROS distribution [43, 44]. Mitochondria are also important for redox homeostasis, with mitochondrial quality control mechanisms, especially mitophagy, being involved. Selective degradation of ROS producing damaged mitochondria prevents excessive oxidative stress and ensures a functional mitochondrial network. Thus, dysregulation of mitophagy can lead to accumulation of dysfunctional mitochondria, increasing ROS levels, and contributing to tumor progression or cell death in a context-dependent manner [45–47]. ROS signaling is spatially and temporally separated, allowing localized oxidative signaling without macromolecular damage. mtROS, cytosolic ROS, peroxisomal ROS and membrane-associated NOX-derived ROS have different roles depending on their site of formation and antioxidant buffering capacity. Tumor subpopulations are characterized by heterogeneous redox microenvironments in terms of oxygen tension, metabolic gradients and organelle-specific antioxidant systems, impacting therapy resistance, metastatic adaptation and ferroptosis sensitivity [48].

In addition to the ETC, emerging evidence suggests that enzymes associated with the tricarboxylic acid (TCA) cycle such as pyruvate dehydrogenase and α-ketoglutarate dehydrogenase are major sources of mtROS. Interestingly, the generation of mtROS is strongly dependent on the availability of metabolic substrates, and different fuels (e.g., glucose, glutamine, fatty acids) have differential effects on the redox balance, due to their effects on NADH/FADH2 generation and electron flux [49, 50]. Cancer cells produce increased ROS due to metabolic changes such the Warburg effect and mitochondrial issues. Mutations in mitochondrial DNA (mtDNA) increase genomic instability and ROS generation [51, 52]. Despite being encased in proteins that are just as protective as those found in nuclear chromatin, mtDNA is especially vulnerable to ROS produced by the respiratory chain. In addition to the aging process and illnesses linked to advanced age, its mutations can cause several disorders. Research points to a connection between mitochondrial genetic malfunction and aging [53, 54]. When nucleosomes that structure DNA within chromosomes are disrupted, the chromatin ceases to be compacted and coiled, resulting in the fragmentation of DNA. Chromatin is crucial for the regulation of gene transcription; thus, alterations in its functional qualities may result in errors that induce mutations [55, 56]. For example, when ROS reacts with nitrogenous bases and deoxyribose sugar in DNA, it causes a lot of oxidative damage, which may lead to mutations, cancer, cell death, necrosis, and genetic diseases [57].

### NADPH oxidase (NOX) enzymes

The NADPH oxidase (NOX) enzyme family is crucial for the active generation of ROS as signaling mediators. NOX isoforms exhibit distinct regulatory mechanisms, tissue distributions, and biological functions in cancer. NOX1, NOX2, and NOX5 primarily produce superoxide, while NOX4 predominantly generates hydrogen peroxide and is unique in being constitutively active, not requiring stimulus-dependent assembly. Beyond serving as a general source of ROS, NOX4 plays a significant role in regulating fibrosis, epithelial-mesenchymal transition (EMT), remodeling of the extracellular matrix (ECM), and the activation of the TME stroma. Increased NOX4 expression has been strongly associated with the activation of cancer-associated fibroblasts (CAFs), transforming growth factor-β (TGF-β) signaling, tumor invasion, and metastatic progression [58–60].

In addition to ROS produced by tumor cells themselves, ROS generated by immune cells such as macrophages, neutrophils, myeloid-derived suppressor cells (MDSCs), and other stromal populations are substantial contributors to redox remodeling within the TME [61]. These ROS are involved in regulating cytokine signaling, immune suppression, angiogenesis, and matrix remodeling, thereby influencing tumor progression and therapeutic responses. In cancer, inflammatory signaling and oxidative stress are interconnected through various factors, including cytokines, growth factors, Toll-like receptor agonists, leptin, and hormones like angiotensin II, which stimulate NOX activity [62, 63].

### Other cellular sources

ROS buildup is also influenced by peroxisomes, cytochrome P-450 enzymes, xanthine oxidase (XO), and endoplasmic reticulum (ER) stress responses. Exposure to environmental carcinogens and oncogenic signaling (such as RAS and MYC) increase oxidative stress in the TME [64, 65]. Numerous studies have demonstrated that ROS, which are essential for controlling tumor genesis, development, and advancement, are more prevalent in cancer cells than in normal cells. It’s interesting to note that ROS is said to have two roles in the formation and management of tumors [66, 67]. On the one hand, by controlling metabolism, proliferation, and angiogenesis, elevated ROS levels provide larger survival advantages. However, ROS are also an effective tool for inhibiting or eliminating cancer cells, which is important during radiation and chemotherapy. By controlling functional proteins such as GPXs, SLC7A11, and PARP-1, ROS-dependent apoptosis, ferroptosis, and autophagy-mediated cell death have been shown to inhibit tumor growth [23, 68, 69]. XO, which catalyzes the oxidative hydroxylation of hypoxanthine to xanthine and xanthine to uric acid, is responsible for the last two stages of purine metabolism in humans. Aging is linked to oxidative stress, immunosenescence, and inflammation, and XO is elevated throughout this time. Endothelial dysfunction, a crucial process that causes a number of age-related illnesses, including cardiovascular diseases (CVDs), appears to be indicated by XO [70–72]. The ER oxirreductin-1 (ERO1) protein is the main source of cellular ROS in eukaryotic cells, which are produced by the ER. A healthy quantity of ROS is necessary for the formation of disulfide bonds, a crucial step in the machinery that folds proteins [73]. ER Ca^2+^ releases can also generate ROS and Redox-sensing thiol groups regulate the ER’s RyR, a Ca^2+^ channel. The RyR can release calcium from the ER by oxidizing its thiol sites [74]. NOX hyperactivity and mitochondrial malfunction, two major biological sources of ROS, are intimately linked to dysregulated calcium transmission. Additionally, the ER expresses the NOX isoform NOX4, which adds to the oxidative stress vicious loop within the ER [42, 75].

### Antioxidant defense systems and redox dependency in cancer

Enzymatic antioxidants like SOD, catalase (CAT), GSH peroxidase (GPx), and peroxiredoxins, as well as non-enzymatic compounds like GSH and vitamins C and E, help cells prevent the buildup of ROS. To preserve redox equilibrium, cancer cells frequently upregulate antioxidant systems through transcription factors like Nrf2 [76, 77]. While it is well known that Nrf2 activation can shield cells from a variety of stresses and toxins, uncontrolled Nrf2 activation has been linked to a number of cancer forms. Cancer cell lines and tumor samples from lung, breast, esophageal, endometrial, and prostate malignancies showed constitutively high Nrf2 levels [78–80]. In fact, there was a negative correlation between Nrf2 levels in the tumor and the prognosis of cancer patients. Increased amounts of Nrf2-target antioxidant proteins, which combat oxidative stress, are responsible for the positive impact of Nrf2 overexpression on tumor growth and survival [81–83]. The main class of antioxidant enzymes that break down superoxide anions is constitutive SOD. Dismutase superoxide is quickly broken down by all types of SOD into the more stable ROS (H_2_O_2_), which is subsequently transformed into water and oxygen by either CAT or GPx. Although it can also be found in the cytosol or mitochondria, the highly effective enzyme CAT is mostly expressed in peroxisomes. Additionally, GPx uses GSH to catalyze the reduction of H_2_O_2_ to water [84, 85]. All cell compartments contain large amounts of GSH, and the primary thiol-based defense mechanism against oxidative and electrophilic stress signals in the cell is the ratio of its oxidized/reduced form (GSH/GSSG). GSSG reduces H_2_O_2_ to H_2_O and O_2_ by giving it an electron. GSH is a cofactor for a number of enzymes, including GPx, which effectively inhibits oxidative damage and, in conjunction with glutaredoxin (Grx), modifies the redox state of proteins by reversible S-glutathionylation [86, 87]. Vitamin C (ascorbic acid) is a powerful water-soluble antioxidant that primarily targets oxygen-centered reactive species, such as superoxide, hydroxyl, and peroxyl radicals. This action helps reduce oxidative damage and lipid peroxidation [88].

**Figure 1:**
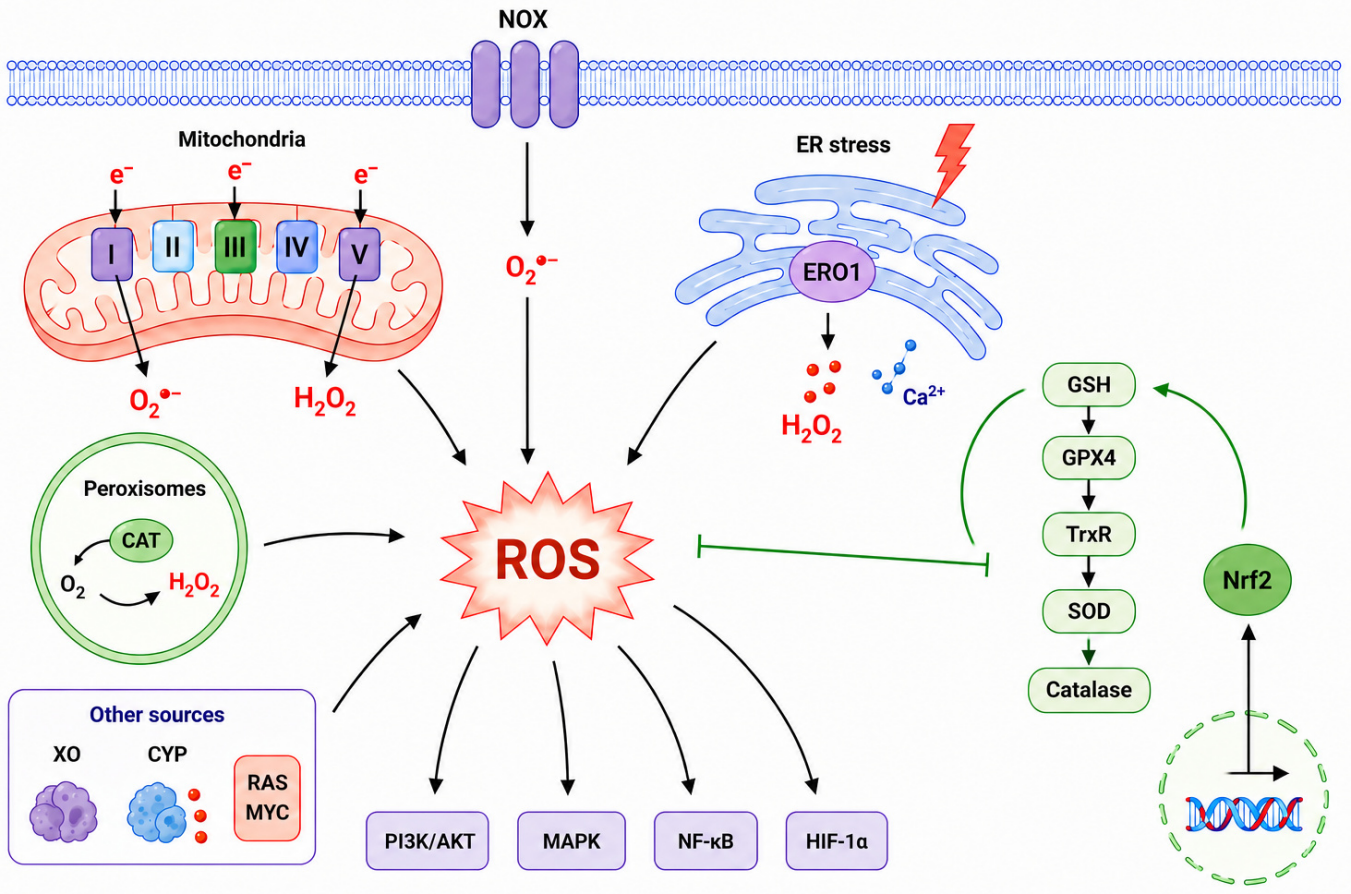
Sources and regulation of reactive oxygen species (ROS) in cancer.

Cancer cells are dependent on the antioxidant systems to keep ROS levels below cytotoxic levels, while maintaining proliferative signaling that is redox-dependent. This is usually termed as “redox addiction”. This increased reliance on GSH, Trx, Nrf2-mediated transcriptional programs and NADPH regeneration pathways allows malignant cells to withstand chronic oxidative stress, hypoxia, metabolic reprogramming and therapeutic insult [89]. But this adaptive antioxidant buffering also provides therapeutic vulnerabilities. Disruption of critical redox defense pathways in many tumors leads to synthetic lethal interactions by selectively overloading the oxidative stress tolerance of cancer cells while sparing normal tissues. Antioxidant systems in cancer are thus not only protective mechanisms but also exploitable metabolic dependencies and key determinants of therapy resistance [90]. Figure 1 shows the main sources and regulation mechanisms of ROS in cancer.

## Oxidative stress and cancer hallmarks

### DNA damage and genomic instability

ROS can cause oxidative DNA damage, including base modifications (e.g., 8-oxo-dG), strand breaks, and mutations that activate oncogenes or inactive tumor suppressors such as TP53. Chronic oxidative stress thus acts as a mutagenic driver of tumorigenesis [91, 92]. Cancer cells are continuously exposed to oxidative DNA damage and are highly dependent on DNA damage response (DDR) pathways for survival. Oxidative lesions such as 8-oxoG are primarily repaired via the base excision repair (BER) pathway through glycosylases including OGG1 and MUTYH, preventing mutagenic G:C→T:A transversions. Persistent oxidative stress induces replication stress characterized by fork stalling, nucleotide pool oxidation, and ssDNA accumulation, activating ATM/ATR signaling and checkpoint responses. Consequently, many tumors rely on PARP-mediated repair and ATR/CHK1 pathways, creating redox-associated synthetic vulnerabilities that can be therapeutically exploited by targeting BER, ATM, or ATR [93, 94]. Cells have many different ways to correct DNA damage. The BER fixes single-strand breaks (SSBs) and oxidized bases, while nucleotide excision repair (NER) resolves worse problems. But excessive ROS can overload these repair pathways or directly harm their function by oxidizing repair enzymes, cofactors, or proteins that are important for finding DNA damage [95]. ROS can impair genomic integrity through epigenetic processes. Oxidative stress can alter DNA methyltransferases and histone-modifying enzymes, causing abnormal methylation patterns and histone acetylation and methylation [96]. These modifications can turn off tumor suppressor genes, activate oncogenes, and modify chromatin structure, making certain regions more vulnerable to DNA damage or copying errors. The epigenetic and chromatin-remodeling effects of ROS contribute to increased genomic instability, resulting in mutagenesis during tumor growth [97].

Besides DNA damage, ROS can affect epigenetic remodeling by the redox-sensitive regulation of chromatin-modifying enzymes. Oxidative stress modulates TET dioxygenases, histone demethylases, DNA methyltransferases, and histone deacetylases, therefore controlling DNA methylation, histone acetylation, and chromatin accessibility. ROS-induced metabolic rewiring influences epigenetic regulation by changing the availability of cofactors such as α-ketoglutarate, acetyl-CoA, NAD^+^, and S-adenosylmethionine. Redox biology directly regulates cancer epigenetics and phenotypic heterogeneity via promotion of transcriptional plasticity, epithelial–mesenchymal transition, stemness, immune evasion, and treatment resistance [98].

### Imbalanced cell proliferation and survival

ROS affects redox-sensitive signaling pathways such as MAPK/ERK, PI3K/AKT, and NF-κB, promoting cell growth and survival. Low to moderate ROS levels activate these cascades, while high ROS cause apoptosis or necroptosis. ROS modulate several cell growth and survival signaling pathways as intracellular second messengers. ROS reversibly oxidize cysteine residues on regulatory proteins, including phosphatases and kinases, altering their activity and downstream signaling [99–101]. For example, ROS-mediated inactivation of MAPK phosphatases sustains activation of MAPKs, which in turn enhance transcription of cyclins, c-Myc, and other cell cycle regulators. This signaling supports G1/S phase transition and promotes the continued proliferation of tumor cells, even under oxidative stress. Persistent ROS signaling thus provides cancer cells with a growth advantage while subtly promoting genomic instability through replication under oxidative conditions [102, 103]. Redox signaling intersects closely with metabolic reprogramming in cancer cells. ROS stabilize hypoxia-inducible factors (HIFs) under non-hypoxic conditions, driving expression of glycolytic enzymes and glucose transporters, which shifts energy metabolism toward glycolysis, which is a hallmark of rapidly proliferating tumor cells [104]. When Nrf2 is activated at the same time, it also increases the expression of antioxidant and metabolic genes, keeping ROS levels in a range that helps cells survive and providing biosynthetic precursors for cell division. These changes work together to give cancer cells both energy and a way to keep their redox levels stable [26, 105].

ROS also regulate the PI3K/AKT pathway, a central axis controlling cell survival, metabolism, and proliferation. Oxidative modification of PTEN, a tumor suppressor phosphatase, at its catalytic cysteine leads to reversible inactivation, resulting in the accumulation of phosphatidylinositol (3,4,5)-trisphosphate (PIP3) and sustained AKT activation. Activated AKT phosphorylates downstream targets such as mTOR, BAD, and GSK3β, which inhibit apoptosis, promote protein synthesis, and enhance metabolic adaptation. This ROS-mediated PTEN inactivation is exploited by tumor cells to survive in high-ROS microenvironments and under nutrient or oxygen limitation, enabling continued proliferation [42, 106].

### Angiogenesis and metastasis

ROS stabilize hypoxia-inducible factor 1-alpha (HIF-1α), upregulating vascular endothelial growth factor (VEGF) and promoting angiogenesis. Oxidative stress enhances EMT and matrix metalloproteinase (MMP) activation, facilitating metastasis. These pro-angiogenic factors stimulate endothelial cell proliferation, migration, and tube formation, supporting neovascularization within the TME. Enhanced vascular networks provide oxygen and nutrients to proliferating tumor cells and facilitate tumor growth, while ROS-mediated modulation of VEGF signaling also primes endothelial cells for sprouting and remodeling [107]. ROS directly influence endothelial dynamics by affecting cytoskeletal organization, cell–cell junctions, and interactions with the ECM. Moderate ROS levels enhance endothelial cell motility, promoting sprouting and extension of nascent vessel networks. However, excessive ROS disrupts adherens junctions and tight junction proteins, increasing vascular permeability. This results in abnormal, leaky tumor vessels, elevated interstitial fluid pressure, and irregular blood flow, which together facilitate tumor progression and create heterogeneous oxygenation conditions that promote selection of aggressive cancer phenotypes [108, 109]. On the other hand, oxidative stress contributes to EMT in cancer cells, a critical step in metastasis. ROS regulate EMT transcription factors such as Snail, Twist, and ZEB1, and modulate the expression of cell adhesion molecules like E-cadherin and N-cadherin. This redox-mediated regulation drives the transition of epithelial tumor cells to a mesenchymal phenotype, increasing motility, invasiveness, and the ability to disseminate from the primary tumor site. By promoting EMT, ROS directly enhances the metastatic potential of tumor cells [110, 111].

In addition to directly promoting EMT, ROS actively remodel the TME to promote invasion and metastatic dissemination. Oxidative stress regulates ECM remodeling through activation of MMPs, collagen crosslinking enzymes and integrin-mediated signaling pathways which contribute to tumor cell motility and tissue invasion [112]. CAFs are major players in this redox-remodeled microenvironment, producing ROS and secreting cytokines, growth factors, and ECM components to support EMT, stromal stiffening, angiogenesis, and metastatic progression. ROS also mediates immune suppression in the TME through tumor-associated macrophages, MDSCs, regulatory T cells and checkpoint signaling pathways that facilitate immune escape during metastatic dissemination [113, 114]. Moreover, oxidative signaling participates in the formation of pre-metastatic niches by promoting the release of inflammatory cytokines, alteration of vascular permeability, metabolic adaptation, and recruitment of stromal and immune cell populations that support secondary tumor colonization. These findings emphasize that ROS-driven metastasis is not just tumor cell–intrinsic but instead reflects complex redox crosstalk between malignant cells and the surrounding microenvironment [115].

### ROS and redox-immune crosstalk in the tumor microenvironment

ROS are a key component of the immunological landscape of the TME. Chronic oxidative stress impacts innate and adaptive immunity and often results in an immunosuppressive environment conducive to tumor development. Tumor-associated macrophages subjected to persistent ROS are often characterized by an M2-like phenotype, including the synthesis of immunosuppressive cytokines and angiogenic and ECM remodeling signals. At the same time, ROS modulate dendritic cell maturation and antigen-presentation capacity, thereby impacting T-cell priming and antitumor immune activation [17, 116]. Oxidative stress induces T-cell dysregulation and exhaustion. Elevated levels of ROS in the TME attenuate T-cell receptor signaling, reduce cytokine secretion, and upregulate the expression of inhibitory checkpoint molecules such as PD-1, TIM-3, and LAG-3. Furthermore, ROS-mediated stabilization of HIF-1α in hypoxia promotes immune escape through up-regulation of PD-L1 expression in tumor and stromal cells. These mechanisms together suppress cytotoxic T-lymphocyte activity and limit efficient antitumor immunity. Importantly, ROS have dual immunological effects depending on concentration, localization, and duration of exposure. Moderate levels of ROS are required for immune cell activation and antimicrobial defense [117, 118].

**Figure 2:**
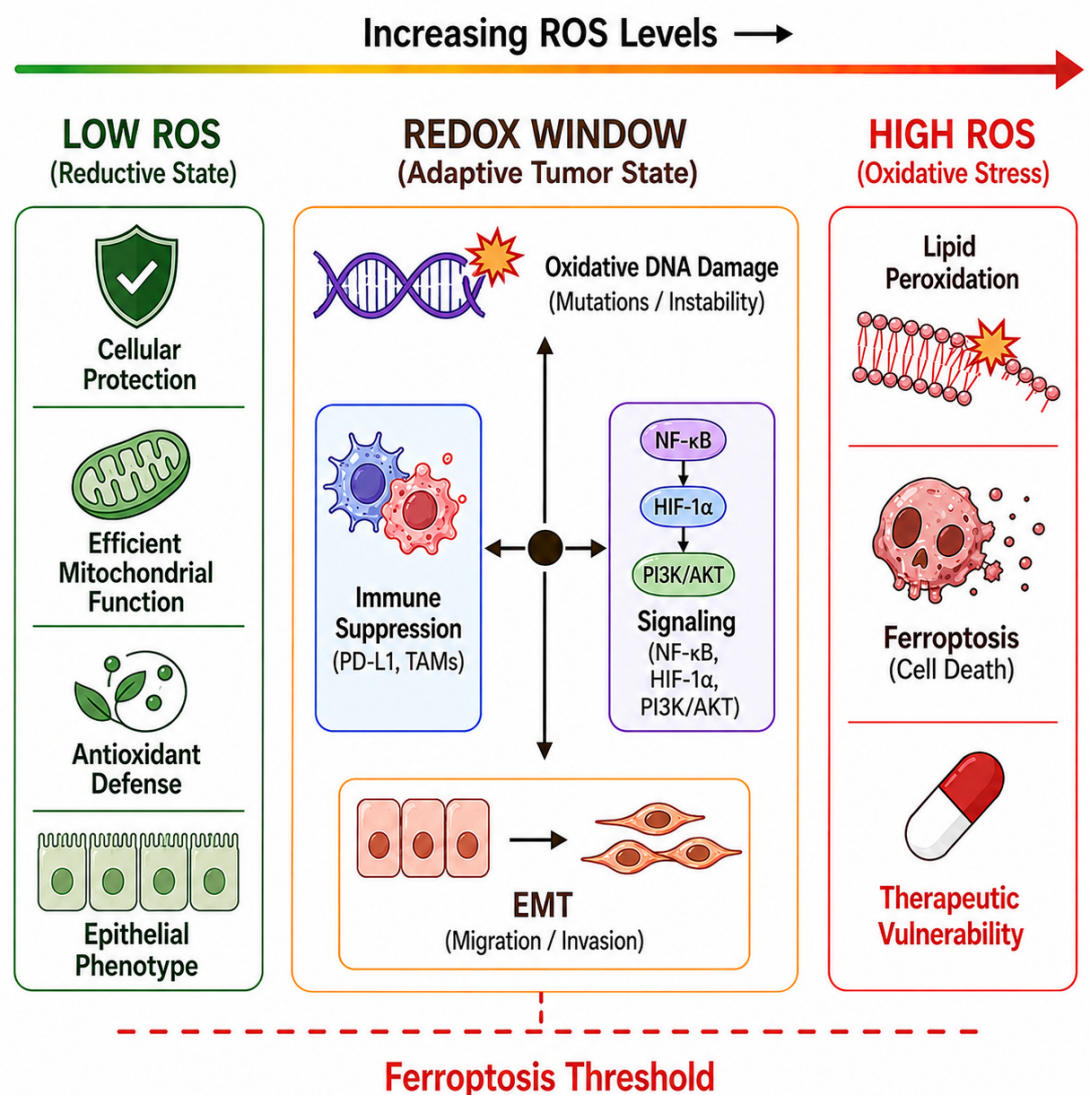
ROS-dependent redox window and its role in cancer hallmarks and ferroptosis vulnerability.

Chronic oxidative stress leads to chronic inflammation, immune suppression, and tumor tolerance. Such threshold-dependent immunological behavior highlights the need for tightly controlled redox modulation strategies in cancer therapy. Moreover, recent evidence also suggests that ROS-mediated immunogenic cell death (ICD) may enhance therapeutic responsiveness via stimulation of dendritic cell activation, antigen cross-presentation, and recruitment of cytotoxic T cells. Therefore, combining ROS-modulating therapies with immune checkpoint inhibitors is a new approach to turn immunologically “cold” tumors into “hot” tumors that are more amenable to immunotherapy [119]. Figure 2 illustrates that cancer cells depend on a tightly controlled ROS “redox window,” where moderate ROS promote tumor progression, but excessive ROS trigger cell death, creating a therapeutic vulnerability.

## Therapeutic targeting of redox balance in cancer

Oncogene activation, metabolic reorganization, and mitochondrial dysfunction cause cancer cells to exhibit elevated basal levels of ROS compared with normal cells. This chronic oxidative stress helps tumors to develop, but it also leaves an intrinsic weakness: Cancer cells are at or near their redox tolerance limit. They are therefore exceptionally sensitive to additional increases in ROS levels. This basic biological difference underpins the redox-based anticancer approach. Redox-based therapeutic interventions against cancer can also be broadly divided into two conflicting yet complementary approaches: (i) pro-oxidant approaches that increase redox levels in the cancer cell to the point of induction of redox-dependent apoptosis; (ii) antioxidant-targeting approaches that disrupt the redox defense mechanisms that cancer cells rely on to survive. ROS modulation, in combination with conventional oncology treatments such as radiotherapy, chemotherapy, and immunotherapy, is a rapidly advancing field in precision oncology [120–122].

Despite strong preclinical rationale, the clinical translation of ROS-targeted strategies has been limited. Large trials of antioxidants have failed to show benefit and, in some cases, increased cancer risk, as seen in the SELECT trial (vitamin E) and β-carotene studies in smokers [123]. Conversely, pro-oxidant therapies have demonstrated only modest efficacy in unselected patient populations, largely due to poor tumor selectivity and dose-limiting toxicity. A key challenge is the adaptive capacity of cancer cells, which upregulate antioxidant systems such as GSH and Trx to buffer oxidative stress and develop resistance [124]. In addition, the lack of reliable pharmacodynamic biomarkers limits the ability to monitor redox modulation and guide therapy. Together, these barriers highlight that ROS-based strategies have not yet achieved broad clinical success and underscore the need for more precise approaches that exploit tumor-specific redox vulnerabilities [125].

### Prooxidant therapies

Pro-oxidant therapeutic approaches exploit the increased ROS susceptibility inherent to malignant cells by further elevating intracellular oxidative stress beyond cytotoxic thresholds. One of the reasons why this approach has been chosen is the relative insensitivity of cancer cells compared to normal cells to an oxidative insult: Due to the already existing elevated levels of basal ROS and reduced antioxidant reserve capacity, exogenous generation of ROS is more easily triggered to cause apoptosis, ferroptosis, or necroptosis in tumor cells but not in normal tissues [121, 126]. A variety of clinically approved chemotherapeutic agents achieve their cytotoxic effects by generating ROS. The semiquinone and quinone forms of doxorubicin are a common anthracycline antibiotic applied in breast, ovarian, and hematologic malignancies, in which redox cycling of the drug gives rise to the formation of superoxide anion radicals and then hydrogen peroxide (H_2_O_2_). Nonetheless, doxorubicin-induced cardiotoxicity, explained by excessive ROS accumulation in cardiomyocytes with insufficient antioxidant protection, is an important clinical problem, which is why the development of tumour-selective delivery methods is necessary [127]. Arsenic trioxide (ATO, As_2_O_3_) which was approved against acute promyelocytic leukemia (APL) acts by dual redox process: On the one hand, it directly inhibits GPx and Trx reductase (TrxR), therefore, preventing the removal of ROS, and on the other, it causes the mitochondrial production of ROS due to interference with the ETC. In APL, ATO is a tumour-killing biological agent that has been shown to achieve complete remission in monotherapy, providing clinical validity for the concept of ROS-mediated tumour killing [120, 128]. Elesclomol (STA-4783) is a small-molecule copper chelator that delivers copper ions to the mitochondrial matrix, where they induce hydroxyl radical generation by a Fenton-like reaction. Preclinical models have demonstrated broad antitumor activity, and a Phase III trial has indicated that it enhances progression-free survival in stage IV melanoma and significantly improves it in patients with normal serum lactate dehydrogenase (LDH), which is strongly associated with greater mitochondrial oxidative metabolism and greater sensitivity to ROS [129]. One of the attractive approaches to drug repurposing is that of disulfiram-copper (DSF-Cu) complexes. The anti-alcohol dependence drug disulfiram forms bis (dithiocarbamate) complexes with copper ions in vivo, thereby inhibiting the 26S proteasome, triggering the p97-NPL4 pathway, and producing intracellular ROS. Several retrospective studies and preliminary clinical trials have reported improved survival rates in cancer patients who used disulfiram throughout chemotherapy. DF-Cu with carboplatin/paclitaxel Phase I/II trial (NCT02671890) is currently ongoing in advanced non-small-cell lung cancer (NSCLC) [130].

New photodynamic therapy (PDT) technologies are a spatially selective form of pro-oxidant modality. PDT includes the use of a tumour-targeting photosensitizer (e.g., photofrin, 5-aminolevulinic acid, temoporfin) and light activation, which delivers energy to molecular oxygen to produce singlet oxygen (^1^O_2_)—A highly reactive non-radical ROS that causes lipid peroxidation, mitochondrial membrane rupture, and immunogenic tumour cell death. PDT has now been approved for the treatment of oesophageal, lung, and skin malignancies. Current studies are directed toward near-infrared-activable photosensitizers with improved tissue penetration to expand their use to solid tumours [99, 131].

### Targeting antioxidant defence systems

An entirely different and complementary mechanism is the conceptual degradation of the antioxidant defence system, whereby cancer cells rely heavily on it to survive under sustained, constitutive oxidative stress. Adaptively, cancer cells overexpress the GSH system, the Trx system, and the Nrf2-Keap1 transcriptional axis, thereby forming targetable redox vulnerabilities. Interference with such protective mechanisms disrupts redox homeostasis, leading to oxidative cell death with selective targeting of cancer cells [132, 133]. The most common intracellular antioxidant is GSH, which is found in millimolar amounts in cancer cells and catalyses the neutralisation of ROS by GPx. The prototypic GSH depletion agent buthionine sulfoximine (BSO) competitively inhibits the rate-limiting enzyme in GSH production, gamma-glutamylcysteine synthetase (gamma-GCS). Preclinical research further showed that BSO and melphalan exhibited striking synergy in childhood neuroblastoma, prompting a Phase I clinical study that revealed the potential of BSO-mediated GSH depletion in pediatric malignancies. More recently, erastin and other system Xc-inhibitors deprive cancer cells of cysteine, the rate-limiting amino acid for GSH synthesis, and inactivate GPX4, resulting in ferroptosis and lipid peroxidation, types of iron-dependent oxidative cell death with therapeutic potential across various tumour types [132, 133]. The main secondary antioxidant pathway is the Trx system, which includes Trx (Trx1/2), TrxR1/2, and NADPH, and has critical functions in DNA synthesis, protein folding, and redox signalling. TrxR1 overexpression in a variety of tumours is associated with resistance to chemotherapy and a negative outcome. Auranofin (Ridaura) is an antirheumatic medication that is a potent, irreversible TrxR inhibitor, achieved by covalent selenocysteine formation at the enzyme’s active site upon gold(I) addition. Auranofin induces apoptosis by increasing mtROS and is in Phase I/II clinical trials for the treatment of chronic lymphocytic leukaemia (CLL), lung, and ovarian cancers. The reduced CAT level in cancer cells makes them more dependent on TrxR than normal cells, thereby providing therapeutic selectivity [133, 134].

Precision redox oncology targets the unique oxidative dependencies of cancer cells. Cancer cells with oncogenic KRAS, MYC amplification, KEAP1 mutations, mitochondrial malfunction, or high basal ROS need GSH, Trx, GPX4, FSP1, and NADPH-regenerating pathways to survive. Damage to compensatory antioxidant pathways may reduce cancer cell tolerance to oxidative stress while protecting normal tissues. GPX4 and FSP1 inhibition depletes GSH, targeting TrxR, while PARP inhibition boosts ROS in DNA repair-deficient malignancies. Context-specific synthetic lethal interactions may improve adaptive resistance and therapeutic selectivity [135–137].

### Ferroptosis and lipid peroxidation-driven therapeutic vulnerabilities

In contrast to apoptosis, ferroptosis is characterized by the accumulation of oxidized PUFA-containing phospholipids that are produced by iron-catalyzed Fenton reactions and ROS-mediated lipid oxidation. Ferroptosis represents an emerging therapeutic target in redox oncology, since cancer cells with an increased oxidative stress and a dysregulated lipid metabolism are often highly sensitive to ferroptotic cell death [138]. GPX4 is a central regulator of ferroptosis sensitivity by detoxifying phospholipid hydroperoxides and preventing lethal accumulation of lipid ROS. Inhibition of system Xc–cystine transport, depletion of GSH, or direct GPX4 inhibition disrupts this protective pathway and induces ferroptosis. Another important player is ACSL4 (acyl-CoA synthetase long-chain family member 4), which enriches cellular membranes with PUFA-containing phospholipids that are highly susceptible to peroxidation and consequently increases ferroptosis sensitivity [139]. Multiple parallel ferroptosis defense systems beyond GPX4 have been recently identified by studies. Ferroptosis suppressor protein 1 (FSP1) reduces coenzyme Q10 (CoQ10) and suppresses lipid peroxidation at the plasma membrane independently of GPX4, whereas dihydroorotate dehydrogenase (DHODH) provides an additional mitochondrial ferroptosis defense mechanism through mitochondrial CoQ reduction. These compensatory antioxidant pathways are important contributors to therapy-refractory tumor ferroptosis resistance [140, 141].

Increased transferrin receptor expression, ferritin degradation, labile iron accumulation, and mitochondrial iron mishandling can potentiate ROS-induced lipid peroxidation and vulnerability to ferroptosis [142]. Conversely, cancer cells can evade ferroptosis through increased antioxidant buffering, Nrf2 activation, lipid remodeling alterations, iron sequestration, or upregulation of FSP1- and DHODH-mediated defense pathways. Ferroptosis is closely related to ROS generation, metabolic reprogramming, and therapy resistance; thus, ferroptosis-inducing strategies are increasingly studied in combination with chemotherapy, radiotherapy, immunotherapy, and nanoparticle-based ROS amplification systems [135].

### Combining ROS modulation with conventional therapies

The combination of ROS modulation and the currently used cancer treatment methods, such as chemotherapy, radiotherapy, and immunotherapy, is one of the most rapidly developing fields in translational oncology. The combination rationalisation behind the use of combinatorics is based on mechanistic synergy: ROS-modulating agents can sensitise tumour cells to traditional cytotoxic therapy, circumvent resistance, and augment immune-mediated tumour elimination [143, 144]. The cytotoxic action of ionising radiation (IR) is due mainly to the formation of hydroxyl radicals through radiolysis of intracellular water that causes irreparable DNA breaks in the form of double-stranded breaks (DSBs) in the replicating tumour cells. Intratumoral hypoxia restricts radiation-generated ROS, leading to radioresistance. Cancer stem cells (CSCs), which are proportionately the major culprits of radioresistance and tumour recurrence, exhibit lower ROS levels due to higher antioxidant expression; targeting this radioresistance pathway could be achieved by targeting CSC antioxidant systems [99, 143]. The most significant transformation of a fundamental model between redox biology and cancer treatment is perhaps the convergence of ROS modulation and cancer immunotherapy. One type of regulated cell death, called ICD, occurs in response to specific pro-oxidant stimuli and is characterised by the surface exposure of calreticulin (ecto-CRT), ATP secretion, a find-me signal, and HMGB1 release, a danger signal. The combined effects of these damage-associated molecular patterns (DAMPs) include stimulation of dendritic cells, cross-presentation of tumour antigens, and activation of cytotoxic T lymphocyte (CTL) responses. The main inducers of ICD, such as anthracyclines, oxaliplatin, cyclophosphamide, and PDT, share a common mechanism: They cause ER stress and ROS production in mitochondria [144, 145].

Oxidative modification of PD-L1 by ROS at cysteine residues may affect its stability, glycosylation, and interaction with PD-1, thereby altering the response to checkpoint inhibitors. The synergistic antitumor activity of pro-oxidant agents, when combined with anti-PD-1 antibodies, has transformed immunologically cold tumours into immunologically hot tumours, thereby enabling checkpoint blockade. Several Phase I/II clinical trials are ongoing to test this combination strategy in a variety of solid tumour histologies [134, 144]. The existing guidelines on consensus have suggested no regular high-dose antioxidant supplementation during radiotherapy and platinum-based chemotherapy until conclusive results from prospective studies are available [99, 145]. However, the immunomodulatory effects of ROS are highly context-dependent and can also drive immune suppression in the TME. Chronic oxidative stress in T cells contributes to T-cell exhaustion by impairing TCR signaling and enhancing the expression of inhibitory receptors (e.g., PD-1), thereby limiting cytotoxic activity. At the same time, ROS supports the proliferation and suppressive activity of MDSCs and induces macrophage polarization to the pro-tumorigenic M2 phenotype. These effects together highlight a dual role of ROS and emphasize the need for precise modulation to improve the efficacy of immunotherapy [146–148].

Nanotechnology platforms can be used to address the selectivity challenges of systemic ROS modulation. Fenton and Fenton-like reactions catalysed by iron oxide nanoparticles and copper-based metal-organic frameworks (MOFs) are deactivated in the acidic, H_2_O_2_-rich TME at interstitial levels, producing highly toxic hydroxyl radicals selectively at the tumour site: a strategy known as chemodynamic therapy (CDT). Nanoplatforms loaded with glucose oxidase (GOx) use intratumoral glucose to catalyse the production of H_2_O_2_ in the tumour, which in turn starves cancer cells by serving as a metabolic substrate by producing hydroxyl radicals through the Fenton reaction. These methods constitute an excellent amalgamation of tumour microenvironment hijacking and targeted ROS amplification, with several nanoformulations undergoing Phase I trials [134, 149]. ROS-responsive drug delivery and prodrug strategies are under investigation to enhance tumor selectivity and reduce systemic toxicity. The high oxidative stress in many cancers makes it advantageous to release therapeutic payloads in ROS-rich tumor locations using ROS-sensitive linkers such as thioketal bonds, boronic esters, or oxidation-sensitive polymers. ROS-activatable prodrugs are oxidized in the TME to exert cytotoxicity. Nanoparticle-based systems for endogenous ROS generation and delivery of chemotherapeutic medicines, ferroptosis inducers, or immunomodulators are prospective precision redox oncology multimodal therapies [150–152].

Despite promise, key translational hurdles remain for CDT and GOx-based nanoplatforms. Their efficacy depends on heterogeneous factors of the TME (e.g., H_2_O_2_ levels and glucose availability), which can limit consistent ROS generation. Therapeutic accumulation is further hampered by inefficient delivery, rapid clearance, and poor tumor penetration. Furthermore, the lack of clinically predictive models and the difficulties of large-scale reproducibility are still barriers to successful clinical translation [153–155]. A summary of redox-targeted therapeutic strategies in cancer is presented in Table 1.

**Table 1:** Summary of redox-targeted therapeutic strategies in cancer.

**Strategy**	**Key agents**	**Mechanism of action**	**Cancer types**	**Clinical status**	**Advantages**	**References**
Pro-oxidant therapy	Doxorubicin, arsenic trioxide (ATO), β-lapachone, elesclomol	Redox cycling; superoxide via mitochondrial ETC disruption; exceeds cytotoxic ROS threshold.	Leukaemia, APL, solid tumours	Approved/Phase I–III	Selective cancer cell killing due to elevated basal ROS	[120]
GSH synthesis inhibition	Buthionine sulfoximine (BSO), erastin, imuthiol	Inhibits γ-GCS; depletes GSH; erastin blocks system Xc^–^ causing GPX4-mediated ferroptosis.	Melanoma, ovarian, neuroblastoma, glioblastoma	Phase I–II	Sensitises drug-resistant tumours; induces ferroptosis	[132, 133]
Thioredoxin system inhibition	Auranofin (TrxR inhibitor), PX-12, PMX464	Covalent TrxR selenocysteine modification; impairs peroxiredoxin recycling; elevates ROS.	Lung, ovarian, colorectal cancer	Phase I–II	Well-tolerated; repurposed gold-based drug	[133, 134]
Nrf2 pathway suppression	ML385, brusatol, trigonelline, halofuginone	Blocks Nrf2-Keap1 axis; reduces NQO1, HO-1, GPx; sensitizes to oxidative damage.	Lung, pancreatic, liver, breast cancer	Preclinical/Phase I	Overcomes chemo-resistance; broad spectrum	[131, 132]
Radiotherapy and ROS modulation	Ionising radiation ± nimorazole, ATO	Hydroxyl radicals via water radiolysis; ROS amplifiers enhance DNA DSB formation.	Head & neck, brain, breast, prostate	Standard of care and trials	Well-established; synergistic with antioxidant inhibition	[99, 143]
Photodynamic therapy (PDT)	Photofrin, talaporfin, 5-ALA, temoporfin	Photosensitizer + light → singlet oxygen (^1^O_2_); oxidizes lipids, proteins, nucleic acids.	Skin, oesophageal, lung, bladder, H&N	Approved/Phase II–III	Tumour-localised; minimal systemic toxicity	[144, 145]
ROS + immunotherapy combination	Anti-PD-1/PD-L1 + pro-oxidant agents	Induces ICD; releases DAMPs (CRT, HMGB1, ATP); activates DCs; enhances checkpoint blockade.	Melanoma, NSCLC, colorectal, bladder	Phase I–III	Converts ‘cold’ to ‘hot’ tumours; broadens IO response	[134, 144]
Nanoparticle-based CDT	Iron oxide NPs, Cu-MOFs, GOx nanoplatforms, TiO_2_-NPs	Fenton/Fenton-like reactions; tumor microenvironment H_2_O_2_ amplification; chemodynamic therapy.	Liver, breast, glioma, lung cancer	Preclinical/Phase I	Spatially controlled ROS; reduced systemic toxicity	[134, 149]
Ferroptosis induction	Erastin, RSL3, sulfasalazine, FIN56	Inhibits system Xc^–^ or GPX4; promotes iron-dependent lipid peroxidation and lethal lipid ROS accumulation.	Pancreatic, lung, liver, glioblastoma, therapy-resistant tumors	Preclinical/early phase	Targets redox-adapted and drug-resistant cancer cells; synergistic with chemotherapy and immunotherapy	[156, 157]
ROS-responsive drug delivery and prodrugs	Thioketal nanoparticles, boronic ester prodrugs, ROS-responsive polymers	ROS-triggered drug release selectively within oxidative tumor microenvironments.	Solid tumors, hypoxic tumors	Preclinical/early phase	Reduced systemic toxicity; enhanced tumor specificity	[34, 134, 149]

## ROS, redox homeostasis, and advanced metabolic reprogramming in cancer

Cancer cell survival under oxidative stress is supported not only by increased glycolysis or pentose phosphate pathway (PPP) flux but also by an integrated and compartmentalized redox metabolic network that ensures precise control of NADPH production, mitochondrial function, and biosynthetic demands. Although the PPP is a major cytosolic source of NADPH, it works in concert with other pathways, including one-carbon metabolism, serine biosynthesis, malic enzyme (ME1/ME2), and IDH1/2, to maintain redox buffering across different cellular compartments. The malate-aspartate shuttle (MAS) is an essential but often ignored system that indirectly regulates redox balance by transferring reducing equivalents between the cytosol and mitochondria. MAS maintains NAD+/NADH ratios, thus supporting continued flux through glycolysis and avoiding reductive stress. This stabilizes metabolic conditions that inhibit excessive mtROS production. At the same time, mitochondrial NADPH pools, mainly produced by IDH2 and nicotinamide nucleotide transhydrogenase (NNT), are functionally separated from cytosolic NADPH, indicating the importance of NADPH compartmentation for protecting organelle-specific redox integrity.

Cancer cells also rewire amino acid metabolism to support antioxidant capacity. Serine and one-carbon metabolism (through SHMT1/2 and MTHFD enzymes) fuels nucleotide synthesis and methylation reactions and produces reducing power via folate-mediated NADPH production. This dual role links redox control to epigenetic regulation and proliferation. Importantly, these pathways are especially relevant under hypoxia or mitochondrial dysfunction, where canonical sources of NADPH are compromised. Glutamine addiction is another major adaptation that sustains both biosynthesis and redox homeostasis. α-Ketoglutarate derived from glutamine feeds the TCA cycle and is reductively carboxylated to citrate via IDH1/2 under hypoxic or ETC-limited conditions to generate lipids while maintaining NADPH homeostasis. At the same time, glutamine metabolism guarantees the maintenance of GSH synthesis, thereby linking nutrient availability to ROS detoxification capacity.

**Figure 3:**
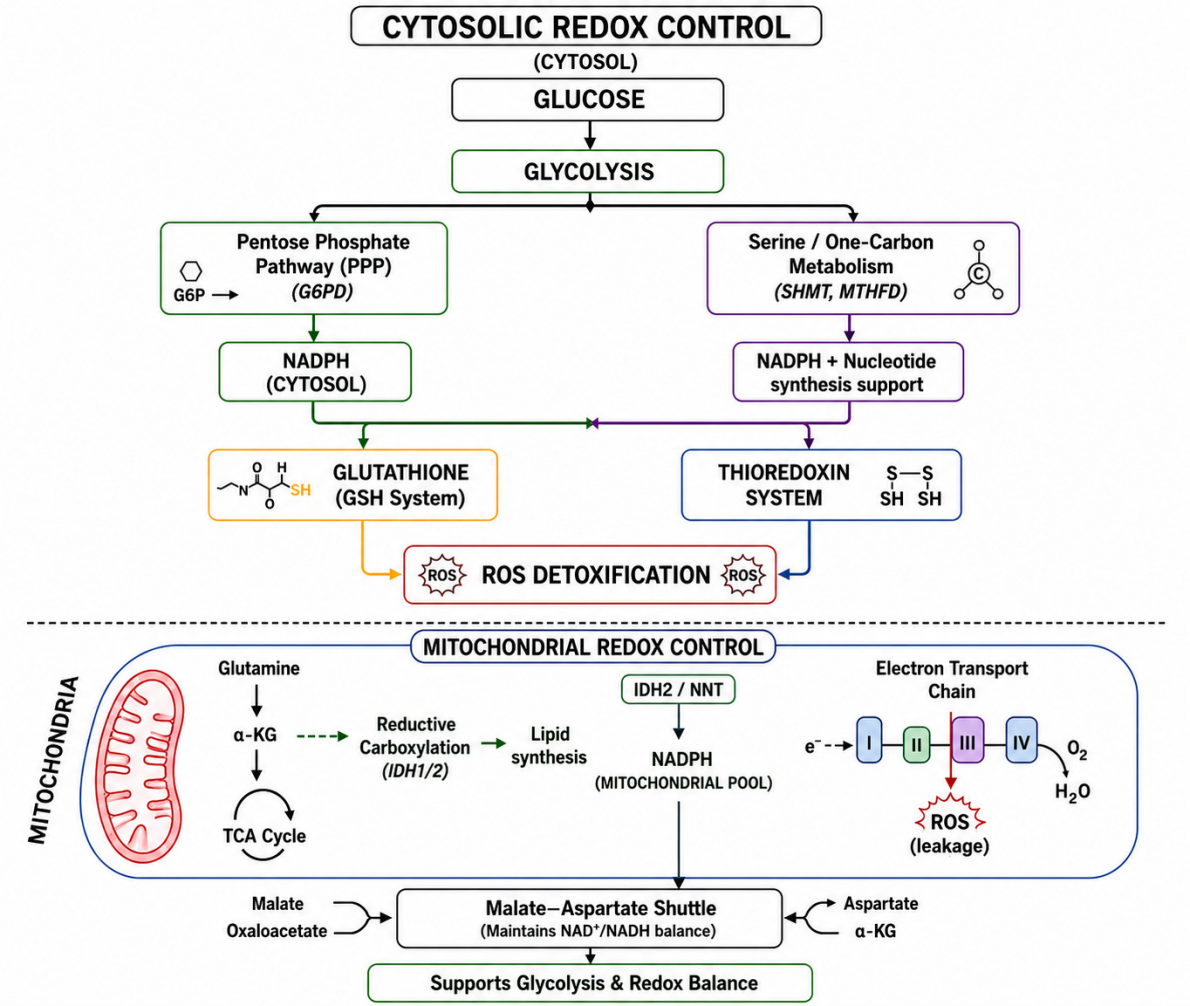
Interconnected metabolic pathways in cancer cells coordinate NADPH production and redox homeostasis, enabling precise regulation of ROS levels to support tumor growth, survival, and resistance to oxidative stress.

Cancer cells do not simply minimize ROS but rather maintain a dynamic redox setpoint, with controlled ROS levels promoting signaling pathways (e.g., PI3K–AKT, HIF-1α) while avoiding cytotoxicity. This balance is attained through metabolic plasticity that remodels carbon flux in response to environmental stress, oncogenic signaling, and mitochondrial function. Disruption of these finely tuned networks, especially the NADPH-producing pathways or glutamine metabolism, results in catastrophic ROS accumulation, exposing key therapeutic vulnerabilities. Thus, cancer metabolism should be considered not as a glycolysis-dominant system but as a multi-layered redox-regulatory architecture where carbon metabolism, electron transfer systems, and antioxidant defenses are tightly integrated to sustain tumor growth under fluctuating oxidative conditions. Cancer cells are able to manage ROS for survival and proliferation because of the interrelated metabolic pathways that produce and transport NADPH to maintain redox equilibrium (Figure 3).

## Challenges and future perspectives

### Defining the therapeutic redox window

The main obstacle that ROS-based cancer treatments face is the need to establish a specific therapeutic redox window that activates programmed cell death in cancer cells while protecting normal tissue from permanent oxidative damage [122]. Elevated basal ROS levels in cancer cells result from their metabolic and signaling defects. In contrast, normal cells maintain their normal ROS levels because both cancer and normal cells need ROS to support vital biological functions, including signal transduction, immune system activation, and tissue repair [158]. The process of systemic ROS modulation poses a significant risk because any increase beyond the toxic threshold will cause complete damage to DNA, proteins, and lipids in healthy cells, resulting in significant side effects and a complete breakdown of cellular balance [23]. ROS function through a complex mechanism that requires specific dosage levels, as low to moderate doses activate pro-tumorigenic pathways that lead to cellular changes and cell survival. In contrast, high-dose levels exceed a cell’s capacity to handle antioxidants, leading to cell death via apoptosis, ferroptosis, and other forms of controlled cell death [159]. The clinical progress of several prooxidant agents has been severely hindered by an exceptionally narrow therapeutic window, which requires high drug concentrations to achieve therapeutic effects but results in dangerous toxicity to patients [122].

### Tumor heterogeneity and spatial redox complexity

Tumorigenesis is a multistep process in which oncogenic mutations arising in a single somatic cell confer clonal advantage as the initiating event; yet mutations alone are insufficient for full malignant transformation, as additional epigenetic alterations and extrinsic environmental pressures including aging, chronic inflammation, and genotoxic insults must converge to drive progression toward an irreversible, highly heterogeneous, and invasive lesion [160]. Tumor heterogeneity is the term for the different kinds of cancer cells that can be found in a tumor (intratumoral) or between patients (intertumoral). These differences can be in genetics, gene expression, and metabolism. This complexity, resulting from clonal evolution influenced by Darwinian selective pressures from both intrinsic cellular factors and extrinsic microenvironmental stimuli such as hypoxia, altered pH, and nutrient gradients, produces phenotypically distinct and drug-resistant subpopulations capable of immune evasion, therapeutic resistance, and sustained uncontrolled growth, rendering treatment, diagnosis, and disease monitoring exceedingly difficult [160, 161]. This tumorigenic cascade is driven by ROS dysregulation, highly reactive oxygen-derived intermediates that balance intracellular levels for normal homeostasis. DNA damage, genomic instability, and oncogenic signaling pathway activation result from oxidative stress, an imbalance between ROS generation and antioxidant defenses. It also provides a pro-tumorigenic milieu that promotes cancer cell proliferation, survival, and metastasis [162]. Redox complexity arises not merely from elevated ROS levels per se, but from the dynamic tension between oxidant production and the adaptive antioxidant machinery that cancer cells co-opt to survive; the Nrf2–Keap1 signaling axis serves as the master regulator of this response, wherein oxidative stress releases Nrf2 from its repressor Keap1, enabling nuclear translocation and transcriptional activation of over 250 cytoprotective genes governing GSH synthesis, Trx activity, and NADPH regeneration thereby establishing an adaptive redox buffer that, while protective in normal tissue, becomes a pro-oncogenic mechanism when constitutively activated in tumor cells [22, 162].

Sustained NrF2 activation in cancer, frequently mediated by loss-of-function mutations in Keap1, enhances GSH synthesis, upregulates detoxifying enzymes, and promotes drug efflux transporter expression, collectively producing a multidrug-resistant phenotype that confounds therapeutic strategies [162]. The resulting redox complexity is further amplified by the heterogeneity of ROS thresholds across distinct tumor subclones, the compartment-specific nature of ROS generation from NOX and mitochondrial ETC sources and the redundancy of parallel antioxidant systems, including both the TrxR (TXNRD1) and GSH reductase (GSR) pathways regulated by NrF2, all of which collectively obscure the precise oxidative switch governing the transition between tumor-promoting and cytotoxic ROS signaling, underscoring the urgent need for real-time redox profiling, subtype-specific intervention strategies, and biomarker-guided combination therapies [162, 163].

### Adaptive redox plasticity and antioxidant resistance

Malignant cells exhibit a profound redox adaptation that enables them to survive chronic oxidative stress by developing an enhanced endogenous antioxidant capacity. This metabolic flexibility renders cancer cells relatively insensitive to additional stress inducers, effectively blunting the therapeutic efficacy of conventional treatments such as chemotherapy and radiation [164]. A cornerstone of this adaptive mechanism is the persistent activation of the Nrf2–Keap1 signaling axis; while Nrf2 is typically targeted for degradation by the Keap1–Cul3 complex under resting conditions, oxidative stress stabilizes the protein, allowing it to translocate to the nucleus [158]. Upregulated GSH biosynthesis and Trx system activity have been directly correlated with cancer aggressiveness and resistance to platinum-based chemotherapy and radiation therapy, as these systems enable tumor cells to neutralize apoptotic signals and maintain redox homeostasis. This targeted disruption of redox adaptation is increasingly viewed as a means to overcome drug resistance in advanced tumors [122, 165].

### Lack of robust and dynamic redox biomarkers

Redox-based oncology translation encounters a significant challenge because it requires clinically verified biomarkers that can measure intracellular ROS levels and metabolic fluxes in real time [158]. Current research often relies on static surrogate markers, including measurement of antioxidant enzyme expression levels (e.g., SOD and CAT) and assessment of oxidative damage product accumulation (e.g., malondialdehyde and 8-oxo-G) [23]. While these are useful for indicating past oxidative stress, they fail to capture the rapid, dynamic redox states and interconversions of specific oxidant species that characterize living tumors [158, 166].

This technical limitation leads to several clinical challenges. For example, Poor Patient Stratification, as it is difficult to identify which patients possess the specific “redox phenotypes” most likely to respond to prooxidant or antioxidant interventions [167]. Researchers face difficulties evaluating tumor redox alterations because they lack access to real-time redox-monitoring technology that would enable more precise measurements. The study shows that nonspecific ROS dyes mislead data interpretation, underscoring the need for better scientific methods, including deep-tissue imaging and mass spectrometry [158, 168].

## Future perspectives: advancing precision redox oncology

Recent progress in redox-sensitive biosensors and genetically encoded fluorescent probes such as FRET-based sensors that monitor metabolic cofactors and caspase activity now enables real-time visualization of ROS dynamics at subcellular resolution [167]. These advanced imaging tools offer unparalleled insights into the rapid fluctuations in tumor-specific redox states, overcoming the historical technical hurdle of relying on static, nonspecific surrogate markers [158]. The integration of single-cell multi-omics, spatial metabolomics, and redox proteomics will soon enable precise mapping of oxidative heterogeneity across diverse tumor ecosystems [169]. This comprehensive mapping is essential for rational therapeutic design, as it accounts for the divergent metabolic phenotypes and ROS dependencies found within a single lesion, particularly in specialized subpopulations like CSCs [158]. Future therapies will rely on personalized redox-targeted approaches based on tumor genotype, metabolism, and microenvironment. Identifying tumor-specific redox phenotypes can guide effective redox-based treatment selection [167, 170]. The rational combination of ROS modulation with chemotherapy, radiotherapy, ferroptosis induction, and immunotherapy has the potential to maximize tumor-selective cytotoxicity while minimizing systemic toxicity [171]. For example, combining ROS-generating agents with immune checkpoint inhibitors or radiotherapy can synergistically overwhelm cancer cells’ adaptive defenses, pushing them toward regulated cell death without harming surrounding healthy tissue [172].

## Conclusion

ROS are no longer viewed as toxic metabolic byproducts but are recognized as dynamic, compartmentalized, and context-dependent regulators of tumor biology. This review emphasizes that ROS operate in a delicate balance, where moderate oxidative signaling is beneficial for tumor initiation, metabolic adaptation, immune evasion, angiogenesis, and metastatic progression, whereas overwhelming oxidative stress can lead to apoptosis, ferroptosis, and other forms of regulated cell death. Thus, the essential challenge of redox oncology is not to indiscriminately destroy ROS but to understand and therapeutically exploit the narrow and heterogeneous redox vulnerabilities that distinguish malignant cells from normal tissues. One important finding to emerge from recent studies is that cancer progression and therapy resistance are driven not only by elevated ROS levels but also by adaptive antioxidant rewiring. Tumor cells have evolved a highly plastic redox-buffering system involving GSH, Trx, NADPH-generating pathways, and constitutive Nrf2 activation that enables them to survive under chronic oxidative stress conditions while sustaining proliferative signaling. This adaptive redox plasticity explains the failure of many antioxidant-based clinical trials, as well as the modest clinical efficacy of several pro-oxidant therapies, in the setting of strong preclinical rationale. Importantly, the field is increasingly recognizing that tumor heterogeneity and spatial redox complexity have profound effects on therapeutic response, as different tumor regions and cellular subpopulations have different ROS thresholds, metabolic dependencies, and ferroptosis sensitivities. We believe that the future of redox oncology will be based on the transition from the generalized modulation of ROS to precision-based therapeutic strategies. Emerging technologies such as single-cell multi-omics, spatial metabolomics, genetically encoded redox biosensors, and real-time ROS imaging are anticipated to set new standards for the characterization and monitoring of tumor-specific oxidative states. Such approaches could allow for biomarker-driven patient stratification and identification of tumors that are selectively vulnerable to redox disruption. One of the most promising directions is the therapeutic induction of ferroptosis via targeting GPX4, system Xc^–^, FSP1, or related antioxidant defense pathways, especially in therapy-resistant and metabolically rewired cancers. Simultaneously, rational combinatorial strategies, including ROS modulation with immunotherapy, radiotherapy, metabolic targeting, and nanotechnology-based delivery systems, may overcome adaptive resistance and improve tumor selectivity. In general, the future success of ROS-based cancer therapy will probably hinge on the definition of individualized therapeutic redox windows, the understanding of dynamic redox adaptation within the TME, and the integration of redox biology into the larger framework of precision oncology. Future therapeutic strategies must not only view ROS as tumor-promoting or cytotoxic but must also exploit the inherent instability of tumor redox homeostasis as a selective and targetable vulnerability in cancer.

## Abbreviations

APL: acute promyelocytic leukemia
BER: base excision repair
BSO: buthionine sulfoximine
CAF: cancer-associated fibroblast
CAT: catalase
CDT: chemodynamic therapy
CSCs: cancer stem cells
DHODH: dihydroorotate dehydrogenase
ECM: extracellular matrix
EMT: epithelial-mesenchymal transition
ER: endoplasmic reticulum
ETC: electron transport chain
FSP1: ferroptosis suppressor protein 1
GOx: glucose oxidase
GPX: glutathione peroxidase
GSH: glutathione
HIF-1α: hypoxia-inducible factor 1-alpha
ICD: immunogenic cell death
MAS: malate-aspartate shuttle
MDSCs: myeloid-derived suppressor cells
MMPs: matrix metalloproteinases
mtDNA: mitochondrial DNA
mtROS: mitochondrial reactive oxygen species
NOX: NADPH oxidase
PDT: photodynamic therapy
PPP: pentose phosphate pathway
ROS: reactive oxygen species
TCA cycle: tricarboxylic acid cycle
TME: tumor microenvironment.
Trx: thioredoxin
TrxR: thioredoxin reductase
VEGF: vascular endothelial growth factor
XO: xanthine oxidase

## Declarations

### Copyright

© The Author(s) 2026.
